# TIPS in Older Adults: Reserve-Based Risk Stratification and Practical Approach

**DOI:** 10.3390/jcm15082928

**Published:** 2026-04-12

**Authors:** Yi He, Yuanyuan Li, Langli Gao, Xiaoze Wang

**Affiliations:** 1 Center of Gerontology and Geriatrics, West China Hospital, Sichuan University, Chengdu 610207, China; 18155@wchscu.edu.cn (Y.H.); 10512@wchscu.edu.cn (Y.L.); 2 West China School of Nursing, Sichuan University, Chengdu 610207, China; 3 Department of Gastroenterology and Hepatology, West China Hospital, Sichuan University, Chengdu 610207, China

**Keywords:** transjugular intrahepatic portosystemic shunt, older adults, cirrhosis, hepatic encephalopathy, frailty, sarcopenia, portal hypertension, risk stratification

## Abstract

The transjugular intrahepatic portosystemic shunt (TIPS) is a cornerstone intervention for complications of portal hypertension, including variceal bleeding and refractory ascites. As the population with cirrhosis ages, clinicians increasingly face the question of whether and how to perform TIPS safely in older adults. We reviewed observational cohorts, registry analyses, and systematic reviews/meta-analyses. Existing evidence does not support chronological age as an absolute contraindication; however, multiple studies suggest that advanced age is associated with higher rates of post-TIPS hepatic encephalopathy (HE), early mortality, and readmissions. These findings underscore the need to shift from a binary “eligible vs. ineligible” paradigm to a structured, actionable framework that addresses modifiable risks and anticipates age-related vulnerabilities. Recent clinical practice guidance emphasizes comprehensive pre-TIPS assessment and vigilant post-procedure care, with specific attention to HE risk factors (e.g., prior HE, hyponatremia, renal dysfunction, sarcopenia) and cardiopulmonary reserve. In this narrative review, we propose an elderly-focused clinical pathway built around a four-domain assessment (Liver–Brain–Body–Heart/Kidney) and a traffic-light risk tiering system to guide patient selection, procedural strategy, follow-up scheduling, and triggered management of HE, cardiac decompensation, and renal dysfunction. This pathway aims to preserve the benefits of portal decompression while reducing preventable complications and improving outcomes that are meaningful to older patients, including functional status and quality of life. This narrative review emphasizes that outcomes after TIPS in older adults are determined not by chronological age alone but by multidomain physiological reserve. The proposed pathway informs patient selection, procedural planning, and early post-discharge monitoring in older adults.

## 1. Introduction

The transjugular intrahepatic portosystemic shunt (TIPS) has become an integral component of management for portal hypertension-related complications in cirrhosis. By creating a low-resistance channel between the portal and systemic venous circulation, TIPS effectively reduces portal pressure, is widely used to control acute or recurrent variceal bleeding, treat refractory or recurrent ascites, and serves as a bridge strategy in awaiting transplantation [1,2]. Over the past two decades, advances in technique, imaging guidance, and particularly the adoption of covered stent-grafts have improved shunt patency and procedural reliability, expanding real-world utilization beyond highly selected populations [3,4]. As a result, many centers now encounter older adults with more comorbidity and more complex medication regimens [5].

This demographic shift directly changes the risk calculus surrounding TIPS. Older patients with cirrhosis frequently present with a convergence of factors that are individually associated with worse post-TIPS outcomes: reduced cognitive reserve and subclinical neurocognitive impairment, sarcopenia and malnutrition, frailty and functional dependency, cardiovascular disease (including occult diastolic dysfunction), and vulnerability to renal injury [6,7,8]. These age-associated phenotypes can amplify the physiologic perturbations induced by TIPS—most notably the abrupt increase in portosystemic shunting of gut-derived neurotoxins (e.g., ammonia) and the hemodynamic redistribution that raises venous return [9,10,11]. Consequently, clinicians increasingly face a practical question: not merely whether TIPS can be performed in an older adult, but whether it can be performed safely and whether the post-procedure period can be managed in a way that preserves both survival and functional status.

Importantly, emphasizing higher vulnerability in older adults should not obscure the potential benefits of TIPS when appropriately selected. In patients with refractory ascites, TIPS can reduce ascites recurrence and the need for repeated large-volume paracentesis, which may translate into better symptom control and patient-reported quality of life. Likewise, in variceal bleeding, effective portal decompression can reduce rebleeding risk and the downstream burden of hospitalization. These potential benefits provide the rationale for a reserve-based approach: the goal is not age-based restriction but identifying older patients in whom the expected clinical gain outweighs the risks of post-TIPS HE and cardio-renal decompensation [1,3].

The available evidence argues against a simplistic “age cutoff” for TIPS: chronological age alone poorly reflects biological vulnerability, yet outcomes do appear to worsen beyond higher age thresholds (often >70 years), with more post-TIPS hepatic encephalopathy (HE), early mortality, and HE-related readmissions [12,13,14]. The commonly used >70-year cutoff point likely reflects how the observational literature has been analyzed (categorical thresholds and selective referral of fitter older candidates), rather than a fixed biological boundary. What appears most consistent is that multidomain vulnerability—cognition, frailty, sarcopenia, polypharmacy, and cardio-renal reserve—clusters more often beyond the seventh decade and amplifies post-TIPS syndromes such as HE and decompensation [6]. While current guidelines provide detailed recommendations on indications and liver-centric assessment, they offer limited operational guidance on geriatric vulnerabilities (frailty, cognition, polypharmacy, and caregiver support). This gap underpins our proposed reserve-based pathway for older adults undergoing TIPS [1,2].

Against this background, a practical framework is needed to translate heterogeneous evidence into actionable, geriatric-informed decision-making for TIPS. Here, we synthesize the available evidence and propose an older adult-oriented clinical pathway that integrates a structured pre-TIPS assessment across four clinically relevant domains: hepatic disease severity, neurocognitive vulnerability, nutritional status and physical function (including frailty and sarcopenia), and cardiopulmonary and renal reserve. Based on these elements, patients are categorized into low-, intermediate-, or high-risk strata, which are then used to guide (i) patient selection and pre-procedure optimization for elective TIPS, (ii) procedural planning with an emphasis on avoiding excessive shunting in high-risk individuals, and (iii) a time-defined follow-up schedule with predefined triggers for escalation focused on the complications most consequential in older adults, namely HE, cardiopulmonary decompensation, and renal dysfunction. We propose a conceptual shift from age-based eligibility to multidomain reserve-based risk assessment, integrating liver severity, neurocognitive vulnerability, sarcopenia–frailty phenotype, and cardiopulmonary–renal reserve into a structured clinical pathway.

In this review, “multidomain physiological reserve” refers to the capacity of integrated organ systems to buffer stressors and maintain function when exposed to an acute perturbation such as TIPS-induced hemodynamic and metabolic shifts. Chronological age is an imperfect proxy for this reserve; instead, reserve is better represented by measurable vulnerability phenotypes spanning hepatic severity, neurocognitive vulnerability, nutritional–musculoskeletal status (frailty and sarcopenia), and cardiopulmonary–renal reserve. Conceptually, this approach is concordant with geriatric medicine: it mirrors the Comprehensive Geriatric Assessment (CGA) framework (multidomain assessment and individualized care planning), incorporates frailty constructs (phenotype-based and deficit accumulation models), and operationalizes them into actionable hepatology decisions before and after TIPS.

## 2. Evidence Landscape: Outcomes of TIPS in Older Adults and Sources of Heterogeneity

### 2.1. Search Strategy

This narrative review aimed to synthesize the available evidence on TIPS outcomes in older adults, using a comprehensive search strategy without adhering to systematic review protocols. We conducted a structured search across MEDLINE, Embase, and Web of Science, applying search terms focused on TIPS, cirrhosis, and older age. The search strategy aimed to include relevant studies and identify key findings without performing a formal quality appraisal or risk of bias assessment. We focused on human studies that reported post-TIPS outcomes such as hepatic encephalopathy, mortality, readmissions, and complications.

For guideline synthesis, we prioritized the most recent comprehensive international guidance for TIPS and portal hypertension and used other authoritative statements as complementary sources when they addressed specific domains (e.g., patient selection, HE prevention, cardiopulmonary assessment). Specifically, we prioritized EASL 2025 TIPS Clinical Practice Guidelines, supported by North American practice-based recommendations and the Baveno VII consensus [1,2,15].

### 2.2. Outcome Signals in Older Adults: Interpretation and Heterogeneity

The evidence base evaluating TIPS in older adults is largely derived from observational studies, including retrospective single-center and multi-center cohorts, registry-based analyses, and a limited number of systematic reviews and meta-analyses [5,12,13,14,16,17]. Across these data, a consistent theme emerges that chronological age alone does not preclude benefit from portal decompression, but advanced age is repeatedly associated with a higher burden of post-TIPS adverse events, particularly HE, early mortality, and readmission. Importantly, the signal is not uniform across all age cutoff points. Several studies report more reproducible differences when comparing patients above and below 70 years, whereas findings are less consistent when “elderly” is defined at 65 years, suggesting that clinically relevant vulnerability may become more apparent at higher thresholds or within specific phenotypic subgroups [5]. In parallel, real-world cohorts indicate that carefully selected older patients can achieve technical success and targeted bleeding control or ascites improvement, yet they remain more likely to require readmission—most commonly due to HE—highlighting that post-TIPS trajectories in older adults are often determined by complications occurring after discharge rather than procedural failure [6,18].

Interpretation of the current evidence requires caution because reported outcomes reflect substantial heterogeneity in case mix and practice patterns. First, indication profiles differ across studies: cohorts enriched for refractory ascites tend to include patients with more advanced circulatory dysfunction, malnutrition, hyponatremia, and renal vulnerability than cohorts dominated by variceal bleeding. These baseline differences can substantially influence post-TIPS endpoints such as early mortality and the likelihood of recurrent hospitalization [13,17]. Second, procedural strategy is variable, including differences in stent type and nominal diameter, the use of underdilation or controlled expansion concepts, and adjunctive variceal embolization or collateral management. Because the intensity of portosystemic shunting is mechanistically linked to HE risk, variability in shunt degree may partly explain why age appears strongly predictive in some datasets but not others [19]. Third, and most importantly for geriatric practice, many studies incompletely capture age-related vulnerability phenotypes—frailty, sarcopenia, baseline cognitive impairment, polypharmacy burden, and availability of caregiver support—which likely mediate the association between age and clinically meaningful outcomes such as falls, delirium-like presentations, medication intolerance, and recurrent admissions [20,21]. Consequently, conventional risk models that include age as a single covariate may be insufficiently informative for older patients because they do not distinguish individuals with preserved physiological reserve from those with multidomain vulnerability.

Recent guidelines emphasize that pre-TIPS assessment should extend beyond the evaluation of liver disease severity alone [1,2,22]. This comprehensive approach is supported by various studies that highlight the importance of considering additional physiological and clinical parameters when assessing patients for TIPS. One critical factor that has emerged is diastolic dysfunction, which has been shown to significantly impact post-TIPS outcomes. A study evaluating the revised 2020 Cirrhotic Cardiomyopathy (CCM) guidelines found that patients meeting the 2020 CCM diastolic dysfunction criteria had a 6.7-fold higher risk of cardiovascular events post-TIPS (OR 6.7, 95% CI 1.8–23.4, *p* = 0.004), compared to those meeting the older 2005 criteria [23]. This suggests that assessing cardiac function, specifically diastolic dysfunction, is crucial in predicting post-TIPS tolerance and should be integrated into pre-TIPS evaluations. Additionally, the utility of non-invasive measures such as spleen stiffness measurement (SSM) in assessing portal hypertension severity offers another dimension to pre-TIPS evaluations. SSM has been shown to correlate positively with hepatic venous pressure gradient and decreases significantly after TIPS, providing a non-invasive means to monitor portal hypertension and potentially guide clinical decisions [24]. This approach could be particularly beneficial for elderly patients, where invasive procedures may pose higher risks. These findings collectively support the notion that pre-TIPS assessments should be comprehensive, encompassing a range of physiological and clinical factors beyond liver disease severity alone.

### 2.3. Interpretation and Limitations

Interpretation of the available literature requires caution. Most studies are retrospective and susceptible to selection bias, as older adults referred for TIPS often represent a highly selected subgroup with better perceived reserve than the general elderly cirrhosis population. Reported outcomes are further influenced by heterogeneity in indication mix (bleeding vs. ascites), procedural strategy (stent type/diameter, degree of decompression, and adjunct embolization), and varying definitions of “older age” (≥65/70/75/80). Moreover, key geriatric determinants (frailty, sarcopenia, baseline cognition, polypharmacy, and caregiver support) are often unmeasured, limiting causal inference and the ability to specify a universal age threshold. Accordingly, our conclusions emphasize directionality (advanced age is associated with higher HE/readmission risk) while advocating reserve-based assessment to individualize decisions rather than applying age cutoffs.

## 3. Mechanisms with Practical Implications

### 3.1. Reduced Cognitive Reserve and Heightened Susceptibility to Neuroinflammation

The relationship between systemic inflammation, neurotoxins, and cognitive decline in older adults is a complex interplay of biological processes that become increasingly significant with age. As individuals age, their ability to tolerate systemic inflammation and neurotoxins diminishes, leading to a higher propensity for cognitive decline and conditions such as overt HE after TIPS. This is supported by a growing body of evidence that highlights the role of inflammation and neurotoxins in exacerbating age-related cognitive deficits.

Systemic inflammation has been identified as a key mediator of age-related cognitive decline. Research indicates that inflammatory biomarkers such as interleukin-6 (IL-6) and tumor necrosis factor-alpha (TNF-α) are elevated in older adults and are associated with cognitive impairments, particularly in processing speed and executive function [25,26]. These inflammatory processes are further implicated in the pathogenesis of neurodegenerative diseases, where they contribute to neuroinflammation and subsequent cognitive decline [27]. The presence of systemic inflammation is also linked to structural brain changes, such as cortical thinning, which precedes cognitive impairment and serves as an early biomarker of neurodegeneration [28].

Moreover, the interaction between systemic inflammation and neurotoxins is critical in understanding cognitive decline in older adults. Studies have shown that environmental neurotoxins, when combined with metabolic dysregulation and inflammation, significantly increase the risk of cognitive impairment [29]. This is particularly relevant in the context of TIPS, where the procedure can exacerbate the effects of neurotoxins due to altered liver function, leading to conditions like overt HE. The diminished resilience of the aging brain to such insults underscores the importance of managing inflammation and neurotoxin exposure to mitigate cognitive decline [30].

**Figure 1:**
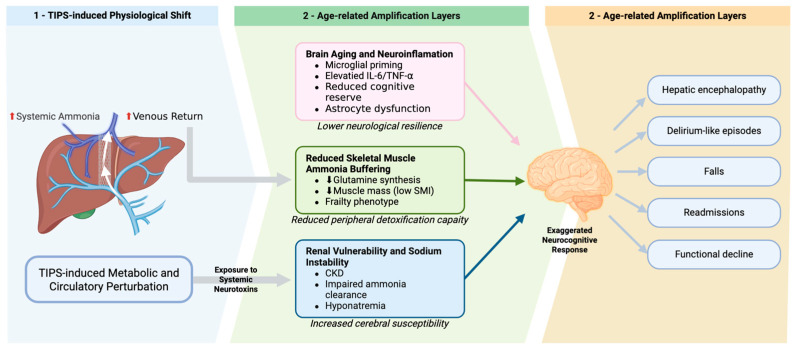
Mechanistic amplification model of post-TIPS hepatic encephalopathy (HE) in older adults. This mechanistic schematic details how underlying age-associated biological vulnerabilities amplify the risk and severity of post-transjugular intrahepatic portosystemic shunt (TIPS) HE in older adults, despite similar initial physiological perturbations. Created in BioRender. (2026) https://BioRender.com/mathl4p (accessed on 16 March 2026). Abbreviations: CKD, chronic kidney disease; HE, hepatic encephalopathy; IL-6, interleukin-6; SMI, skeletal muscle index; TIPS, transjugular intrahepatic portosystemic shunt; TNF-α, tumor necrosis factor-alpha.

The cumulative impact of these factors is evident in various studies that demonstrate the association between systemic inflammation and cognitive decline across different populations and settings. For instance, higher levels of inflammatory markers are consistently associated with poorer cognitive performance and an increased risk of cognitive decline over time [31,32]. Additionally, the role of inflammation in cognitive decline is further supported by evidence linking it to changes in brain morphology, which mediate cognitive performance [33]. These findings collectively highlight the critical need for targeted interventions that address inflammation and neurotoxin exposure to preserve cognitive function in older adults (Figure 1).

### 3.2. Sarcopenia and Malnutrition: Reduced Extrahepatic Ammonia-Buffering Capacity

The role of skeletal muscle in ammonia detoxification is a critical aspect of understanding the metabolic challenges faced by patients with cirrhosis, particularly following procedures such as the transjugular intrahepatic portosystemic shunt (TIPS). Skeletal muscle acts as an auxiliary organ for ammonia detoxification, especially when liver function is compromised. This capacity is significantly impaired in the presence of sarcopenia, a condition characterized by the loss of muscle mass and function, which is prevalent in cirrhotic patients. The reduction in muscle mass associated with sarcopenia can lead to a decreased peripheral buffering capacity for ammonia, exacerbating the risk of complications such as hepatic encephalopathy post-TIPS.

Ammonia-lowering therapies can partially reverse the effects of hyperammonemia on skeletal muscle, thereby improving muscle mass and function. A study by Dasarathy et al. showed that ammonia-lowering treatments, such as L-ornithine L-aspartate, can significantly reduce blood and muscle ammonia levels, increase lean body mass, and improve muscle strength and diameter in hyperammonemic models [34]. This suggests that maintaining or enhancing muscle mass through such therapies could mitigate the adverse effects of sarcopenia on ammonia detoxification capacity. Furthermore, the relationship between muscle mass and ammonia levels is highlighted in studies examining body composition changes post-TIPS. For instance, a study by Merli et al. found that improvements in skeletal muscle index and muscle attenuation were associated with reduced plasma ammonia levels and a lower incidence of hepatic encephalopathy [35]. This underscores the importance of muscle mass not only in physical strength but also in metabolic functions such as ammonia detoxification. Additionally, the use of branched-chain amino acids (BCAAs) has been explored as a therapeutic strategy to enhance muscle ammonia metabolism. BCAAs provide carbon skeletons for the formation of alpha-ketoglutarate, which can combine with ammonia to form glutamine, thus facilitating ammonia removal [36,37]. This mechanism highlights the potential of nutritional interventions to support skeletal muscle function and ammonia detoxification in cirrhotic patients. Thus, the interplay between skeletal muscle and ammonia detoxification is a critical consideration in the management of cirrhotic patients, particularly those undergoing TIPS. Addressing sarcopenia through targeted therapies and nutritional interventions can enhance the peripheral buffering capacity for ammonia, thereby reducing the risk of hepatic encephalopathy and improving clinical outcomes. The evidence supports a multifaceted approach that includes ammonia-lowering therapies, nutritional support, and potentially exercise interventions to maintain or improve muscle mass and function in the older patient population.

### 3.3. Frailty: A Second Axis of Risk Beyond MELD

Frailty indices have emerged as critical tools in predicting mortality in older patients with advanced liver disease, particularly in the context of liver transplantation and the consideration of TIPS [38,39]. The Liver Frailty Index (LFI), in particular, has been shown to enhance mortality risk prediction beyond traditional models such as the MELD score [40]. The study by Lai et al. demonstrated that the LFI provides additional prognostic value over MELD 3.0 alone, highlighting its utility in refining mortality predictions in patients with advanced liver disease [41]. This finding is supported by a meta-analysis that confirmed frailty as a reliable prognostic predictor of outcomes in cirrhotic patients, underscoring the importance of incorporating frailty assessments into clinical practice to improve patient management and outcomes [42].

The predictive power of the LFI is further evidenced by its association with waitlist mortality in liver transplant candidates. A study identified optimal LFI cutoffs that predict waitlist mortality at various intervals, demonstrating that the addition of LFI to MELD-Na scores significantly improves the discriminative ability for mortality prediction [43]. This is corroborated by research indicating that even small improvements in LFI are associated with meaningful reductions in waitlist mortality, suggesting that interventions targeting frailty could be beneficial in this patient population [44]. These findings collectively advocate for the integration of frailty assessments in the pre-transplant evaluation process, potentially influencing decisions regarding TIPS procedures. Moreover, the applicability of frailty indices extends beyond mortality prediction to encompass broader clinical outcomes. For instance, the LFI has been associated with the risk of hepatic decompensation and unplanned hospitalizations in patients with compensated cirrhosis, providing additional prognostic insights that are not captured by traditional liver disease severity scores [45,46]. This reinforces the notion that frailty assessments can offer a more comprehensive evaluation of patient health, guiding clinical decision-making in complex cases such as those involving TIPS. Therefore, the integration of frailty indices, particularly the LFI, into the management of advanced liver disease represents a significant advancement in predicting patient outcomes. By enhancing mortality prediction and offering additional prognostic information, frailty assessments are increasingly considered in the pre-procedural evaluation for TIPS and liver transplantation. The evidence supports the routine use of frailty indices to improve patient stratification and optimize clinical outcomes in this vulnerable population.

### 3.4. Limited Cardiac and Renal Reserve

The hemodynamic changes induced by TIPS can lead to significant clinical challenges, particularly in older patients with limited cardiac and renal reserve. The procedure increases venous return and alters systemic hemodynamics, which can precipitate decompensation in susceptible individuals [47,48]. This is particularly evident in patients identified as high-risk by the Freiburg Index of Post-TIPS Survival (FIPS), who are more likely to experience further decompensation and acute-on-chronic liver failure (ACLF) after TIPS, leading to reduced transplant-free survival [49]. The increase in venous return and preload can exacerbate cardiac decompensation, especially in elderly with diastolic dysfunction, which has been identified as a significant predictor of post-TIPS cardiac complications [50]. This underscores the importance of pre-procedural cardiac assessment to identify patients at risk of heart failure following TIPS.

Moreover, the systemic hemodynamic changes post-TIPS can also impact renal function. The procedure’s effect on central blood volume and venous return can lead to renal decompensation in patients with limited renal reserve and collectively contribute to the alleviation of splanchnic vascular congestion and restoration of central hypovolemia [47,51]. The impact of TIPS on renal function is multifaceted. A study investigating the role of renal function on HE following TIPS placement for refractory ascites found that patients with chronic kidney disease or those on hemodialysis were at an increased risk for HE post-TIPS [52]. This suggests that renal impairment can exacerbate the neurological complications associated with TIPS, highlighting the need for careful pre-procedural assessment and post-procedural monitoring of renal function in these patients.

## 4. A Clinical Pathway for TIPS in Older Adults

### 4.1. Referral Triage and Indication Alignment

TIPS should be considered in older adults based on the same core indications applied to the general cirrhosis population, most commonly recurrent or refractory variceal bleeding and refractory or recurrent ascites. In older adults with cirrhosis, TIPS decision-making should be treated as a peri-procedural “care pathway” rather than a single technical act, because outcomes are frequently driven by post-discharge syndromes (HE, cardiopulmonary decompensation, and renal dysfunction) rather than by shunt patency alone [6]. A retrospective study of 83 patients with refractory ascites found that functional independence was associated with reduced 1-year mortality (OR 0.22, 95% CI 0.05–0.77, *p* = 0.02), highlighting the importance of pre-procedural geriatric assessment [53]. A propensity score-matched study of 106 elderly patients found that TIPS brought 30-day mortality of 24% vs. 12% in younger patients, *p* = 0.19) but higher HE-related readmissions (28% vs. 10%, *p* = 0.04) [54]. The elderly patients with CCM require caution that a prospective study of 107 patients found that CCM patients had lower post-TIPS portal vein pressure (16.7 ± 4.4 vs. 18.9 ± 4.8 mmHg, *p* = 0.022) but similar 1-year survival (13.2% vs. 4.3%, *p* = 0.093) [55]. These data support TIPS as a viable option for elderly patients when selected carefully, with functional status and comorbidities guiding decision-making.

A minimum pre-TIPS evaluation in older adults should include liver disease severity (MELD/MELD-Na, Child–Pugh, and bilirubin/INR/creatinine/sodium), infection/inflammatory status, and prior decompensations, while deliberately extending beyond liver metrics to characterize biological reserve. This geriatric extension should cover baseline neurocognitive vulnerability (prior overt HE; screening for covert HE where feasible; review of deliriogenic medications), sarcopenia/frailty and nutrition (CT-based muscle indices when available, or bedside function measures), and cardio-renal reserve (ECG and comprehensive echocardiography with attention to diastolic function; pulmonary hypertension screening; baseline kidney function and recent AKI trajectory). Frailty tools and sarcopenia assessment add value because they capture extrahepatic vulnerability that MELD does not, and they are clinically actionable through prehabilitation (nutrition, mobility, and medication simplification) prior to elective procedures [40,56].

### 4.2. Risk Stratification That Directly Maps to Actions

**Figure 2:**
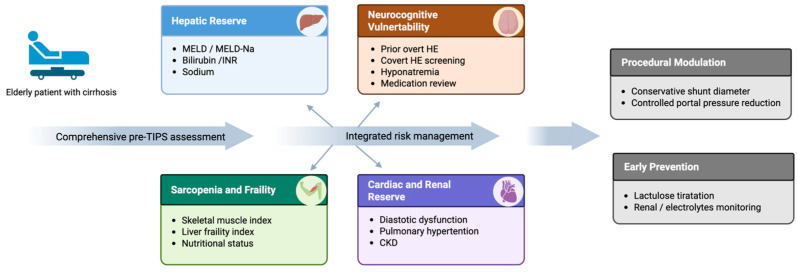
Geriatric-oriented clinical pathway for transjugular intrahepatic portosystemic shunt (TIPS) in older adults. This comprehensive schematic outlines a proposed multi-stage clinical pathway designed to optimize TIPS delivery and safety in older adults (≥70 years) with cirrhosis. Created in BioRender. (2026) https://BioRender.com/8ioy0mo (accessed on 16 March 2026). Abbreviations: ACLF, acute-on-chronic liver failure; CKD, chronic kidney disease; HE, hepatic encephalopathy; INR, International Normalized Ratio; LFI, Liver Frailty Index; MELD, Model for End-Stage Liver Disease; SMI, skeletal muscle index; TIPS, transjugular intrahepatic portosystemic shunt.

Risk stratification in older adults should be explicitly linked to “what we do differently,” rather than used as a passive prognostic label. A practical approach is to classify patients into low/intermediate/high risk based on the convergence of: hepatic reserve, neurocognitive vulnerability, sarcopenia, frailty, and cardio-renal reserve. Prognostic tools can support—but should not replace—clinical synthesis. For example, the FIPS was developed to predict post-TIPS survival and can identify higher-risk patients who may experience subsequent decompensation [49,57]; in older adults, such tools are best used to trigger intensification of optimization and follow-up rather than as absolute exclusion criteria. The proposed clinical pathway (Figure 2) integrates multidomain assessment, reserve-based risk stratification, and individualized procedural planning to guide decision-making in older adults undergoing TIPS.

### 4.3. Operational Definitions and Suggested Tools

To facilitate implementation across diverse practice settings, we operationalize each assessment domain in the proposed pathway using commonly available tools, while emphasizing that no single instrument is mandatory. For frailty and physical function, we recommend the Liver Frailty Index (LFI) when feasible because it is liver-specific and performance-based; when LFI is not available, validated alternatives such as the Fried frailty phenotype or the Short Physical Performance Battery can provide pragmatic estimates of functional reserve. For sarcopenia, CT-based quantification at the L3 level (skeletal muscle index) is preferred when cross-sectional imaging is already obtained for routine TIPS planning; if CT-derived measures are unavailable, psoas-based indices or bedside surrogates (e.g., grip strength) can be used as screening measures to flag vulnerability phenotypes that warrant intensified counseling and follow-up. For neurocognitive vulnerability, we prioritize clinical history of prior overt HE and recommend screening for covert HE where feasible using established tools such as PHES or app-based psychometric tests (e.g., Stroop/EncephalApp), acknowledging that these tests vary by region and language; when HE-specific tools are not practical, a pragmatic baseline cognitive screen (e.g., MoCA) may be considered to document cognition and support post-TIPS change detection, rather than to diagnose HE. For cardiopulmonary and renal reserve, we recommend routine ECG and comprehensive echocardiography with explicit assessment of diastolic function and right-sided parameters, complemented by pulmonary hypertension screening when clinically suspected; clinically significant cardiac dysfunction or pulmonary hypertension should trigger heightened caution, multidisciplinary review, and consideration of non-TIPS alternatives. Renal reserve is operationalized by baseline eGFR and recent creatinine trajectory, together with serum sodium as a marker of circulatory dysfunction; worsening creatinine or progressive hyponatremia in the early post-discharge period should trigger rapid reassessment of volume status, infection, medication effects (diuretics/laxatives), and early escalation of supportive therapy. Across domains, we frame these parameters as pragmatic triggers to guide optimization and monitoring intensity, rather than as universal exclusion thresholds, given heterogeneity in indications, procedural strategies, and local resources.

### 4.4. Procedural Planning in Older Adults

Mechanistically, greater portosystemic diversion increases systemic ammonia load and reduces first-pass hepatic clearance. Although no universal “safe” post-TIPS portal pressure gradient (PPG) threshold exists for the elderly, clinical practice increasingly favors avoiding excessive PPG reduction in high-HE-risk phenotypes. Contemporary EASL guidance emphasizes individualized decompression rather than fixed PPG targets, particularly when HE risk is substantial [1]. Importantly, shunt reduction remains an effective rescue strategy. In an interventional series of patients undergoing TIPS reduction for refractory HE, clinical improvement occurred in approximately 70–90% of cases after successful diameter reduction, supporting the concept that shunt intensity directly influences neurocognitive toxicity [58].

## 5. Hepatic Encephalopathy: The Central Complication in Older Adults

### 5.1. Incidence and Age-Dependent Risk Gradient

Hepatic encephalopathy (HE) is the most common complication following TIPS and the principal driver of post-procedural readmissions. In the general cirrhotic population, overt HE occurs in approximately 25–50% of patients after TIPS placement [1,2]. However, multiple cohort studies demonstrate a higher incidence in advanced age, particularly beyond 70 years. In a retrospective cohort of cirrhotic patients ≥70 years, Adlakha and Russo reported overt HE in 45% of elderly patients compared with 28% in younger controls, with significantly higher HE-related readmissions (28% vs. 10%, *p* = 0.04) [54]. Similarly, Bisht et al. observed increased HE incidence and early mortality in older individuals undergoing TIPS [12]. A systematic review and meta-analysis by Ahmed et al. evaluating TIPS outcomes in elderly patients found that advanced age was associated with increased post-TIPS HE and short-term mortality, though heterogeneity across studies reflected differences in indication and technique [5]. Importantly, Saad et al. demonstrated that older age independently predicted early mortality after TIPS, suggesting that HE may act as a mediator of adverse outcomes in this population [14]. Collectively, these data indicate that age ≥70 represents a clinically meaningful inflection point, but age alone incompletely explains risk variability.

### 5.2. Beyond Chronological Age: A Multidomain Vulnerability Model

Age functions primarily as a surrogate for clustered vulnerabilities. Post-TIPS HE risk is amplified when advanced age coexists with prior overt HE, hyponatremia, chronic kidney disease, sarcopenia, frailty, and polypharmacy. The FIPS incorporates age as a prognostic variable and identifies patients at risk for further decompensation and ACLF after TIPS [57,59,60,61]. However, frailty and sarcopenia are not directly captured in most prediction models, despite their strong association with outcomes [40,42,62]. Thus, a shift from “age-based” to “reserve-based” risk stratification is warranted in older adults.

### 5.3. Pathophysiological Amplification in the Aging Brain

TIPS increases systemic ammonia exposure by bypassing hepatic clearance. The degree of PPG reduction correlates with shunt flow intensity. Smaller-diameter or controlled-expansion stents have been proposed to mitigate excessive shunting and reduce HE risk [19]. While no universal safe PPG threshold exists, overly aggressive decompression increases neurological toxicity—particularly in vulnerable patients.

Aging is associated with chronic low-grade systemic inflammation, which lowers neuronal resilience. Elevated IL-6 and TNF-α levels correlate with domain-specific cognitive decline in older adults [25,26]. Neuroinflammatory signatures and microglial activation have been implicated in cognitive deterioration [27,30]. Hyperammonemia interacts with this inflammatory milieu, exacerbating astrocytic dysfunction and neurotransmission impairment [10]. Thus, in older patients, identical ammonia elevations may produce disproportionately severe neurocognitive consequences compared with younger individuals.

### 5.4. Sarcopenia: Collapse of Peripheral Ammonia Buffering

Skeletal muscle serves as a major extrahepatic ammonia detoxification organ via glutamine synthesis. Kumar et al. demonstrated that ammonia-lowering reverses sarcopenia-related proteostasis impairment in cirrhosis models [34]. Gioia et al. showed that improvement in skeletal muscle index after TIPS was associated with reduced plasma ammonia and lower HE incidence [35]. Branched-chain amino acids enhance muscle ammonia metabolism [37].

In older adults, sarcopenia is highly prevalent and reduces buffering capacity, creating a mechanistic pathway by which insufficient muscle detoxification and sustained hyperammonemia contribute to overt HE. This positions sarcopenia not merely as a frailty marker but as a pathophysiological determinant of HE risk.

### 5.5. Renal Dysfunction and Sodium Disturbance as Synergistic Drivers

Renal dysfunction further limits ammonia clearance and destabilizes electrolyte homeostasis. Zhao et al. demonstrated that chronic kidney disease significantly increased HE risk following TIPS placement for refractory ascites [52]. Hyponatremia independently increases cerebral susceptibility to osmotic shifts and encephalopathy. In older adults, renal vulnerability is compounded by diuretic exposure, infection risk, and reduced effective arterial blood volume. Thus, renal function must be considered a dynamic HE amplifier.

### 5.6. Prevention Strategies in High-Risk Older Adults

Given the high incidence of post-TIPS HE in elderly cohorts, preventive strategies should be proactive. Recommended components include conservative shunt diameter selection in high-risk phenotypes, early lactulose titration with bowel movement targets, consideration of rifaximin in recurrent-risk patients, and early renal and electrolyte monitoring. Such structured bundles may reduce HE-related readmissions, which disproportionately affect older patients.

For recurrent or refractory HE despite optimized medical therapy, shunt reduction is effective. Sarwar et al. reported clinical improvement in approximately 70–90% of patients undergoing TIPS reduction for refractory HE [58]. In elderly patients with repeated admissions and functional decline, earlier consideration of shunt modification may be appropriate—balancing the risk of recurrent portal hypertensive complications.

Taken together, post-TIPS HE in older adults reflects the interaction between increased ammonia load and reduced systemic buffering capacity. Recognition of sarcopenia, renal dysfunction, and cognitive vulnerability as modifiable contributors provides opportunities for targeted risk mitigation.

## 6. Cardiac Decompensation and Renal Dysfunction: Hemodynamic Stress Testing of Limited Reserve

### 6.1. Hemodynamic Redistribution After TIPS

While hepatic encephalopathy dominates neurological outcomes after TIPS, cardiocirculatory and renal complications represent the second major axis of post-procedural morbidity in older adults. Unlike HE, which is largely neuro-metabolic, these complications arise from abrupt hemodynamic redistribution and increased venous return. In elderly patients with limited cardiac and renal reserve, TIPS functions physiologically as a “circulatory stress test,” often unmasking subclinical dysfunction. Pitton et al. showed that right atrial pressure rises significantly within the first week after TIPS and influences portal hemodynamics [9]. In younger cirrhotic patients with preserved cardiac reserve, these changes may be tolerated. In contrast, older adults frequently exhibit diastolic dysfunction, reduced ventricular compliance, pulmonary hypertension, and subclinical right ventricular impairment. Thus, TIPS may precipitate overt heart failure or pulmonary congestion in this subgroup.

### 6.2. Diastolic Dysfunction

Cirrhotic cardiomyopathy (CCM) is characterized by impaired contractile responsiveness and diastolic dysfunction. The revised 2020 CCM criteria refined diagnostic thresholds [63]. Bommena et al. demonstrated that patients meeting 2020 CCM diastolic dysfunction criteria had a 6.7-fold higher risk of cardiovascular events post-TIPS (OR 6.7, 95% CI 1.8–23.4, *p* = 0.004) [23].

Similarly, Schneider et al. found that pre-TIPS diastolic dysfunction was independently associated with post-TIPS cardiac decompensation [50]. Notably, Liu et al. reported that patients with CCM had a similar 1-year survival compared with non-CCM patients, but exhibited altered portal hemodynamics and higher risk of early instability [55]. These findings suggest that diastolic dysfunction does not always increase mortality directly, but it significantly increases early morbidity and decompensation. In older adults, age-related myocardial stiffening compounds CCM-related impairment, making diastolic reserve a critical screening parameter.

### 6.3. Renal Dysfunction: A Parallel Axis of Risk

Renal outcomes after TIPS are complex and bidirectional. On one hand, portal decompression may improve renal perfusion in selected patients with refractory ascites by restoring effective arterial volume. On the other hand, abrupt circulatory changes and cardiac dysfunction may precipitate acute kidney injury (AKI). Busk et al. demonstrated that systemic hemodynamic changes after TIPS significantly alter renal function parameters [48]. Zhao et al. found that chronic kidney disease significantly increased the risk of post-TIPS hepatic encephalopathy, underscoring the interaction between renal impairment and neurological complications [52]. Roy & Kulkarni emphasized the complexity of ascites management in patients with concomitant renal dysfunction, highlighting the delicate volume balance required after portal decompression [51]. In elderly patients, renal vulnerability is amplified by baseline CKD, diuretic exposure, infection risk, sarcopenia, and reduced renal autoregulatory capacity. Thus, even modest hemodynamic shifts can precipitate AKI.

## 7. Future Directions: Toward a Geriatric-Informed TIPS Paradigm

The expanding use of TIPS in aging cirrhotic populations challenges traditional, liver-centric decision models. While existing guidelines emphasize hepatic severity and procedural technique, emerging evidence suggests that post-TIPS outcomes in older adults are predominantly determined by systemic reserve rather than portal pressure metrics alone. Several critical knowledge gaps and research priorities warrant attention.

### 7.1. From Age-Based to Biology-Based Risk Stratification

Chronological age is an imprecise surrogate for physiological vulnerability. Current prediction models (e.g., MELD, MELD-Na, FIPS) incorporate age but do not capture multidomain geriatric phenotypes such as frailty, sarcopenia, baseline cognitive reserve, or polypharmacy burden. Future prospective studies should integrate validated frailty metrics (e.g., Liver Frailty Index) and CT-based sarcopenia indices and quantify medication-related cognitive risk. A biologically informed composite model could improve discrimination for post-TIPS HE, cardiac decompensation, and functional decline beyond current scores.

### 7.2. Prospective Trials of Prehabilitation Before Elective TIPS

Sarcopenia and frailty are potentially modifiable. However, no prospective trials have evaluated structured prehabilitation programs before elective TIPS in older adults. Future interventional studies should test short-term nutritional optimization and protein-calibrated diets with BCAA supplementation. Primary endpoints should include not only mortality and HE incidence, but also functional independence, quality-of-life scores, and readmission rates. This shift aligns outcome assessment with geriatric priorities.

### 7.3. Procedural Modulation: Individualized Portal Decompression

The optimal degree of portal decompression in elderly patients remains undefined. Although smaller-diameter or controlled-expansion stents are conceptually attractive for reducing HE risk, high-quality comparative data in elderly cohorts are lacking. Randomized or registry-based comparisons of 8-mm vs. 10-mm stents or 6-mm vs. 8-mm stents in patients ≥70 years old should be warranted. Evaluation of staged or incremental shunt expansion strategies should also be explored. Precision modulation of shunt intensity may represent a key strategy for balancing portal control and neurological safety.

### 7.4. Multidisciplinary Care Models

Emerging evidence suggests that multidisciplinary care pathways integrating hepatology, geriatrics, cardiology, nutrition, and rehabilitation expertise may improve outcomes in patients with advanced liver disease. Multidisciplinary cirrhosis clinics have been associated with improved guideline adherence, reduced unplanned hospitalizations, and more comprehensive management of frailty and nutrition-related complications [64]. In older adults being considered for TIPS, such collaborative care models may facilitate more accurate assessment of physiological reserve, optimization of modifiable risk factors such as sarcopenia and medication burden, and coordinated post-procedural monitoring [65].

Future research should evaluate structured multidisciplinary care pathways specifically for older patients undergoing TIPS. Such models may include geriatric assessment before elective procedures, integrated cardiology evaluation for patients with suspected cirrhotic cardiomyopathy, and early rehabilitation or nutritional support programs. Implementation studies examining whether multidisciplinary management reduces HE-related readmissions, functional decline, and health-care utilization would provide important insights for optimizing care in this growing patient population.

## 8. Conclusions

In older adults—particularly those >70 years—post-TIPS HE, early mortality, and readmissions are more common, yet age alone should not be used as an absolute contraindication. The future of TIPS in elderly patients lies in transitioning from liver-centered metrics to multidomain reserve assessment, from reactive complication management to proactive vulnerability mitigation, and from survival-focused endpoints to function-centered outcomes. TIPS in older adults should not be approached as a simple procedural decision, but rather as a multidimensional assessment of systemic reserve and vulnerability.

## Data Availability

No new data were created or analyzed in this study.

## Abbreviations

The following abbreviations are used in this manuscript:

ACLF	Acute-on-chronic liver failure
AKI	Acute kidney injury
BCAA	Branched-chain amino acids
CCM	Cirrhotic cardiomyopathy
CKD	Chronic kidney disease
CT	Computed tomography
EASL	European Association for the Study of the Liver
HE	Hepatic encephalopathy
HVPG	Hepatic venous pressure gradient
IL-6	Interleukin-6
INR	International normalized ratio
LFI	Liver Frailty Index
MELD	Model for End-Stage Liver Disease
MELD-Na	Model for End-Stage Liver Disease—Sodium
PPG	Portal pressure gradient
SMI	Skeletal muscle index
SSM	Spleen stiffness measurement
TIPS	Transjugular intrahepatic portosystemic shunt
TNF-α	Tumor necrosis factor-alpha
